# Anti-obesogenic potential of curcumin, disulfiram, and their synergistic effects in HFD-fed obese Swiss albino mice

**DOI:** 10.3389/fendo.2026.1954754

**Published:** 2026-10-15

**Authors:** Jitendra Kumar Patel, Raghunath S. More, Alakh Narayan Singh, Anurag Gupta

**Affiliations:** 1 Department of Anatomy, Institute of Medical Sciences, Banaras Hindu University, Varanasi, Uttar Pradesh, India; 2 Department of Microbiology, Institute of Medical Sciences, Banaras Hindu University, Varanasi, Uttar Pradesh, India

**Keywords:** anti-obesogenic, curcumin, diabetes, disulfiram, high-fat diet, obesity, repurposing of drugs

## Abstract

**Aims:**

This study aimed to investigate the preventive anti-obesogenic effects of disulfiram and curcumin, including their potential synergistic effects, in a high-fat diet-induced obese and diabetic mouse model by evaluating body weight gain, food intake, glycaemic control, lipid profile, insulin and leptin levels, liver injury markers, and histopathological changes. Diabetes and obesity are dual pandemics affecting millions of people worldwide. Curcumin, commonly used in Indian kitchens, and the repurposed drug Disulfiram, previously used for other purposes, are evaluated to assess their anti-obesogenic properties.

**Materials and methods:**

Inducing obesity and diabetes through an HFD in Swiss albino mice provides a suitable strategy for testing the preventive effects of supplements or drugs against obesity. To achieve this, we established five groups: one fed a control diet, one fed an HFD, and three treatment groups that received curcumin, Disulfiram, or both simultaneously by oral gavage. We measured food intake, weight gain, the Lee obesity index, lipid profiles, liver injury enzymes, insulin and leptin levels, and liver histopathological changes.

**Results:**

These results suggest that treating high-fat diet (HFD)- fed mice with either Curcumin or Disulfiram alone, or combining both simultaneously, for 12 weeks effectively controls weight gain, improves glucose metabolism, reverses abnormal lipid profiles, and reduces Insulin and Leptin resistance.

**Conclusion:**

The combination treatment with Curcumin and Disulfiram shows a synergistic effect and a significantly enhanced anti-obesogenic effect. However, treatment with Curcumin alone also shows greater effectiveness compared to Disulfiram alone.

## Introduction

The recent demographic data illustrates that globally 890 million individuals are living with obesity (1) and 589 million aged 20 to 79 years with diabetes (2), underscoring the coexistence of dual metabolic pandemics. This crisis is mainly driven by Western dietary culture, characterized by overconsuming calories from high-fat and processed foods. The current understanding of obesity highlights that it involves chronic, low-grade tissue inflammation rather than the simple accumulation of adipose tissue. This inflammation is a major driver of insulin resistance, which further progresses to diabetes. Studies also indicate that nearly 197 million people suffer from impaired glucose tolerance, a condition heavily linked to obesity (3). To decipher the complexities of obesity and diabetes and to test preventive and therapeutic interventions, the High-Fat Diet (HFD) mouse model serves as the benchmark because it closely mimics human metabolic alterations (4). In murine models, administering an HFD containing 45% or 60% kilocalories from fat elicits a predictable set of biochemical alterations (5). High lipid intake causes both adipocyte hyperplasia and hypertrophy, triggering the secretion of pro-inflammatory cytokines such as Tumour Necrosis Factor-alpha (TNF-α) and Interleukin-6 (IL-6) (6). This pathology also alters lipid profiles and liver markers, specifically SGPT, SGOT, HDL, and LDL levels, ultimately resulting in Non-Alcoholic Fatty Liver Disease (NAFLD) (7). Furthermore, these inflammatory reactions impair pancreatic beta-cells, compromising insulin production (8). Beyond insulin, the regulation of other crucial metabolic hormones, such as leptin and adiponectin, is severely disrupted during obesity and diabetes (9).

Disulfiram (DSF), historically known as Antabuse, is an FDA-approved drug for treating chronic alcoholism that functions by inhibiting aldehyde dehydrogenase (ALDH) (10). Recent studies demonstrate that Disulfiram reduces food intake, limits body weight gain, and decreases blood glucose levels (11) in HFD-fed mice (12, 13). It also improves glucose tolerance and insulin sensitivity in cell culture by activating autophagy (14). Because ALDH1A1 is a key enzyme involved in weight regulation and energy homeostasis during retinoic acid biosynthesis, Disulfiram facilitates weight loss by inhibiting this specific pathway (15). Proving its formulation versatility, poly-ϵ-caprolactone (PCL) nanoparticles loaded with Disulfiram successfully limit the expansion of white adipose tissue *in vitro* (16). Additionally, DSF suppresses chronic inflammation and insulin resistance by regulating the NLRP3 (nucleotide-binding domain, leucine-rich–containing family, pyrin domain–containing-3) inflammasome (17).

Curcumin, the primary bioactive component of turmeric, possesses phenolic hydroxyl groups that confer potent antioxidant properties, protecting the liver and pancreas during obesity and diabetes (18). Recent research shows that Curcumin’s activation of AMP-activated protein kinase (AMPK) promotes glucose uptake in muscles, stimulates fatty acid oxidation, and inhibits gluconeogenesis in the liver (19).

This preclinical study was conducted to investigate and evaluate pharmacological interventions with potential therapeutic effects on obesity- and diabetes-related outcomes. The purpose of the study is to identify a combined treatment approach that enhances the effectiveness and cost-efficiency of existing or commonly available therapies for human metabolic disorders.

## Material and methods

Forty Swiss albino mice, weighing 23 ± 2 g and aged 6–8 weeks, were obtained from the Central Animal House, Institute of Medical Sciences, Banaras Hindu University, Varanasi. They were housed in a well-lit, air-conditioned departmental animal house facility (Department of Anatomy, IMS, BHU) with food and tap water supplied ad libitum. After one week of adaptation, the mice were weighed and randomly assigned to five groups (n = 8 mice/group) for the following 12-week treatments: Group 1 (Control) – mice fed a normal diet; Group 2 (HFD) – mice fed a high-fat diet (60% calories from fat); Group 3 (HFD+ Cur) – mice fed a high-fat diet and treated with Curcumin at 90 mg/kg body weight (SRL) (20); Group 4 (HFD+ DSF) – mice fed a high-fat diet and treated with Disulfiram at 200 mg/kg body weight (Dr. Morepen Laboratories Ltd.) (14); and Group 5 (HFD+ Cur+ DSF) – mice fed a high-fat diet and treated with Disulfiram simultaneously with Curcumin.

Food intake was determined by weighing the remaining food at the end of each week. Body length was measured with Vernier callipers, and body weight was recorded using a weighing balance. The Lee Obesity Index was calculated using the formula: Lee Obesity Index = [∛(Body Weight (g)/Body Length (cm)) × 1000] (21). Fasting blood glucose levels were measured at 0-, 8-, and 12-week intervals, and an oral glucose tolerance test (OGTT) was performed during the 12th week using an ACCU-CHEK^®^ Instant S glucometer by collecting blood from the tail vein. Thereafter, the mice were sacrificed, blood was collected in red-top vials, and serum was separated via centrifugation at 2500 RPM for 10 minutes. Insulin, leptin, liver function parameters (including lipid profiles), and SGPT and SGOT levels were measured in the serum, whereas HbA1c levels were measured in the plasma. The HOMA-IR (Homeostatic Model Assessment of Insulin Resistance) index was calculated using the formula: HOMA-IR = Fasting Blood glucose (mg/dL) × Insulin (ng/mL)/405 (22). Serum insulin levels were measured by the Architect Chemiflex method, and leptin levels were measured by ELISA. Triglycerides, cholesterol, SGPT, and SGOT were quantified using Coral Clinical Systems kits. For histological analysis of the liver, tissue samples from perfused mice were collected and post-fixed in 10% neutral buffered formalin for 72 hours. Thereafter, the tissues were dehydrated in ascending grades of alcohol, cleared in xylene, embedded in paraffin wax blocks, and sectioned at a thickness of 5–6 µm using a microtome. Hematoxylin and Eosin (H&E) staining was performed, and images were captured using an Olympus BX63 microscope.

### Data analysis

All the Data analyses were performed by GraphPad Prism 8.0.2. Ordinary one- way or two- way analysis of variance (ANOVA) was performed as required by the data sets, followed by multiple comparison tests done by Tukey’s test. The data were represented as mean ± standard deviation (SD). Statistical significance is defined by **** p < 0.0001, *** p < 0.001, ** p < 0.01. The normality of the data was checked by the Shapiro-Wilk test, and homogeneity of variances was checked by the Brown-Forsythe ANOVA test provided within GraphPad Prism. The data successfully passed the normality test (alfa = 0.05) and provided a tightly clustered 45-degree diagonal QQ plot, whereas the Brown-Forsythe ANOVA test showed no significant difference among means (p > 0.05).

## Results

### Curcumin and disulfiram administration reduces food consumption and body weight gain

Tracking the feed consumption behavior across different groups (**Figure 1a**) reveals that HFD-fed mice treated with curcumin or disulfiram consumed significantly less food from the 5th week onwards. At the end of 12 weeks, their consumption was 16.76 ± 0.36 g (p = 0.005) and 17.68 ± 0.88 g (p = 0.013), respectively, compared to 21.61 ± 0.69 g for the normal chow-fed control and 23.54 ± 0.47 g for the HFD-fed mice. No significant difference was observed between the Control and HFD groups (p = 0.057), which exceeds the significance threshold (p = 0.05). Notably, the HFD-fed mice treated simultaneously with curcumin and disulfiram showed even lower food intake than the control group (p = 0.022). By week 12, this combination treatment group had an estimated feed consumption approximately 32% lower than the HFD-only group.

**Figure 1:**
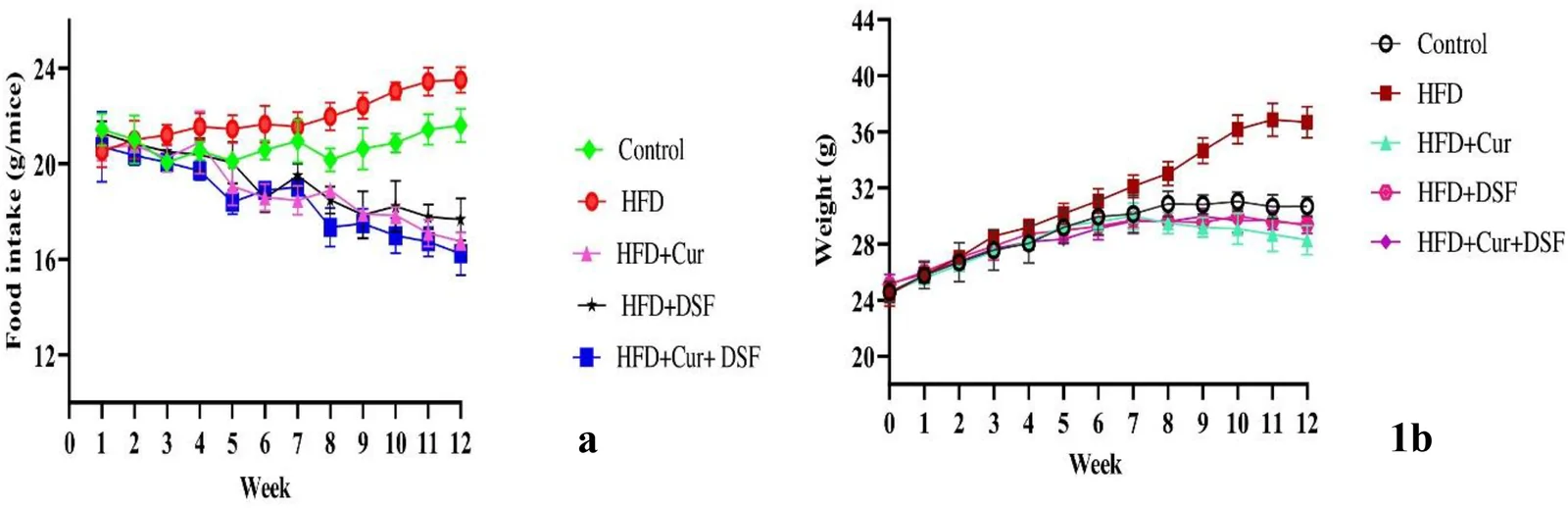
**(a)** Illustrates the food intake behavior, and **(b)** shows the change in weight gain in mice during the 12 weeks of treatment.

A well-established model of HFD-induced obesity was observed in the HFD-fed group (**Figure 1b**), which exhibited a significant increase in body weight compared to the control group from the 7th week onwards. At the end of the 12-week treatment period, the HFD group reached a mean weight of 36.70 ± 1.12 g (p = 0.001), which was significantly higher—by 39.75%—than the Control mean of 30.70 ± 0.71 g. Supplementing HFD-fed mice with curcumin or disulfiram effectively mitigated this HFD-induced weight gain. From the 8th week onwards, the HFD+Cur group maintained a significantly lower weight than the Control group, reaching a mean of 28.28 ± 1.04 g (p = 0.007) at the 12th week. Disulfiram alone also prevented weight gain, maintaining a stable weight of 29.40 ± 0.53 g from the 5th to the 12th week of treatment. The combination treatment (HFD+Cur+DSF) demonstrated the most consistent weight management, stabilizing around 29.32 ± 0.57 g. Overall, the HFD+Cur group showed a steady decline in weight gain, achieving the highest numerical weight reduction at 22.94%, followed by HFD+Cur+DSF at 20.11% and HFD+DSF at 19.89%, whereas the HFD-only group showed a continuous upward trend in weight gain through the 12th week.

Furthermore, none of the three treated groups—HFD+Cur at 309.05 ± 0.96 (p = 0.0002), HFD+DSF at 308.40 ± 0.72 (p < 0.0001), and HFD+Cur+DSF at 309.34 ± 0.34 (p < 0.0001)—showed a Lee obesity index >310 (**Figure 2a**). This suggests that these interventions are highly effective at preventing weight gain compared to the HFD-only fed group, which presented an elevated Lee obesity index of 313.65 ± 0.54 (p < 0.001) relative to the control index of 311.19 ± 1.35 (p = 0.0150).

**Figure 2:**
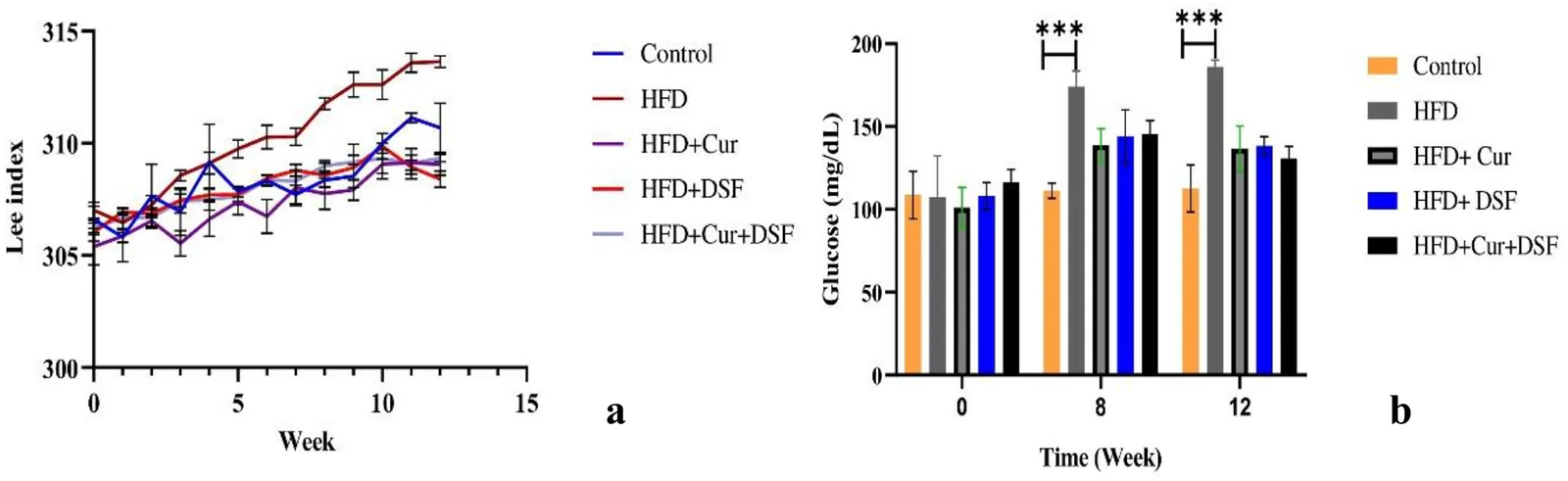
**(a)** Shows the Lee obesity index for the 12 weeks of treatment duration, and **(b)** shows fasting blood glucose levels in mice at 0, 8 and 12 weeks. ***=p < 0.0001. already provided in the data analysis section.

### Role of curcumin and disulfiram in glycemic control and glucose tolerance in HFD-induced obese diabetic mice

At the end of the 12th week, the fasting blood glucose level (**Figure 2b**) in the HFD-fed mice was 230.00 ± 20.00 mg/dL, which is significantly higher than that of the control group (112.67 ± 14.29 mg/dL; p < 0.0001).

The glycated hemoglobin (HbA1c) level (**Figure 3**) in the HFD group (6.55 ± 0.53 mmol/mol) showed a greater than two-fold increase compared to the Control group (3.10 ± 0.30 mmol/mol; p < 0.0010), confirming the induction of long-term hyperglycaemia and type 2 diabetes. Monotherapy with either Curcumin or Disulfiram alongside HFD provided significant protection against these HFD-induced effects. The Curcumin group (5.94 ± 0.81 mmol/mol) indicated slightly better protective effects compared to the Disulfiram group (5.75 ± 0.86 mmol/mol), whereas the HFD+Cur+DSF combination group (4.68 ± 0.51 mmol/mol; p < 0.0311) showed a much more pronounced improvement, bringing the levels closest to the Control. This demonstrates a powerful synergistic effect in preventing obesity-linked diabetic progression.

**Figure 3:**
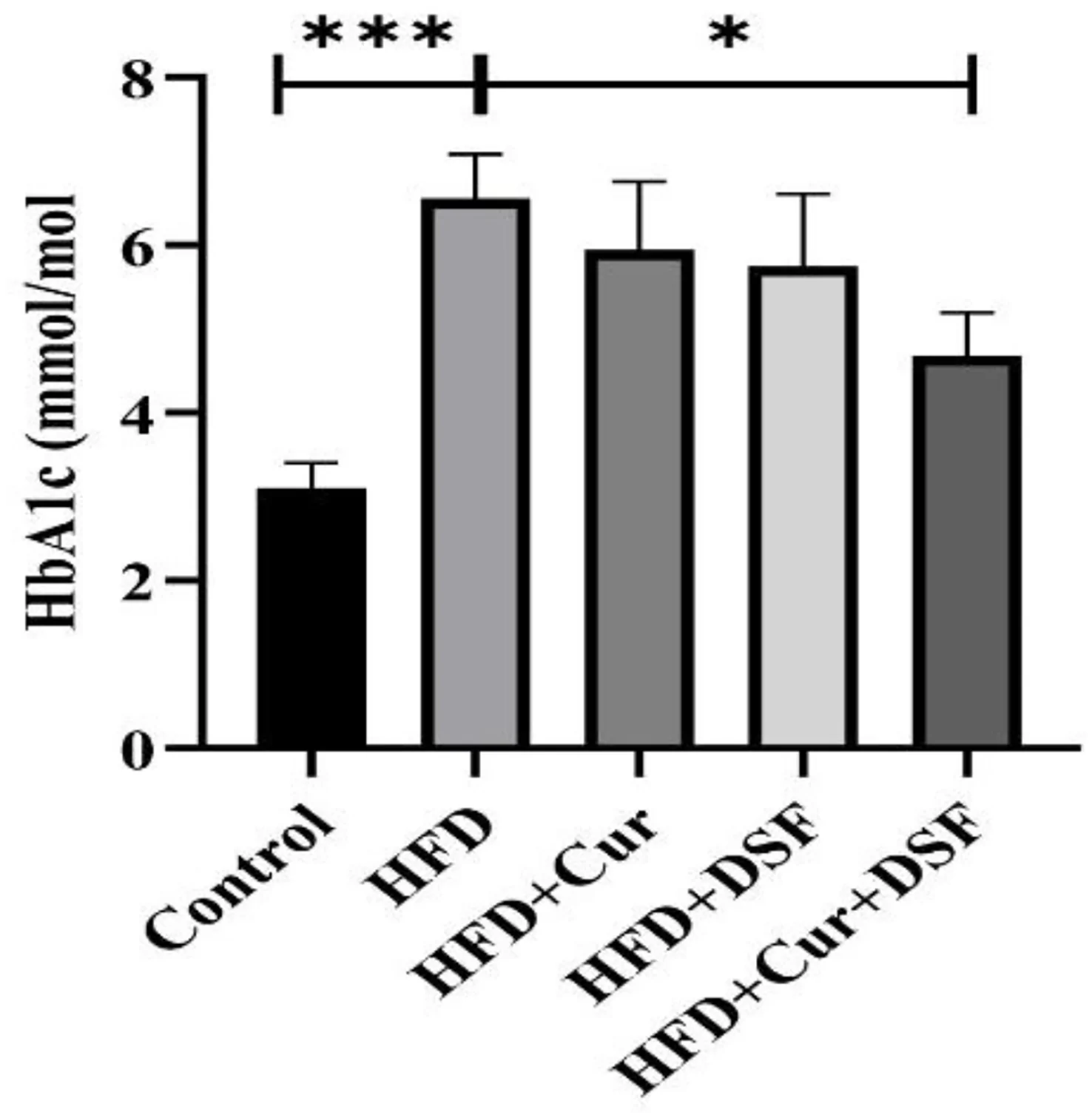
Shows the HbA1C level in various treated groups after the 12^th^ week of treatment. ***=p < 0.0001 and * =p < 0.05.

The oral glucose tolerance test (OGTT) (**Figure 4a**) in the HFD group revealed severely impaired glucose clearance. The baseline glucose level of the HFD group (198.33 ± 8.33 mg/dL) was nearly double that of the Control group (103.33 ± 7.09 mg/dL) and remained highly elevated (206.33 ± 17.62 mg/dL) even after 120 minutes. The Area Under the Curve (AUC) (**Figure 4b**) for the HFD group increased by nearly 54% compared to the Control group, clearly confirming a state of glucose intolerance. Individual treatments with Curcumin and Disulfiram significantly lowered glucose levels at the 120-minute mark to 108.67 ± 6.11 mg/dL (p = 0.0008) and 107.67 ± 6.03 mg/dL (p = 0.0008), respectively, compared to the HFD group. Meanwhile, the HFD+Cur+DSF group peaked at 15 minutes (165.00 ± 12.12 mg/dL), cleared glucose faster to reach its lowest point at 123.00 ± 3.61 mg/dL (p = 0.0013) by 120 minutes, and maintained the most stable overall profile among the treatment groups.

**Figure 4:**
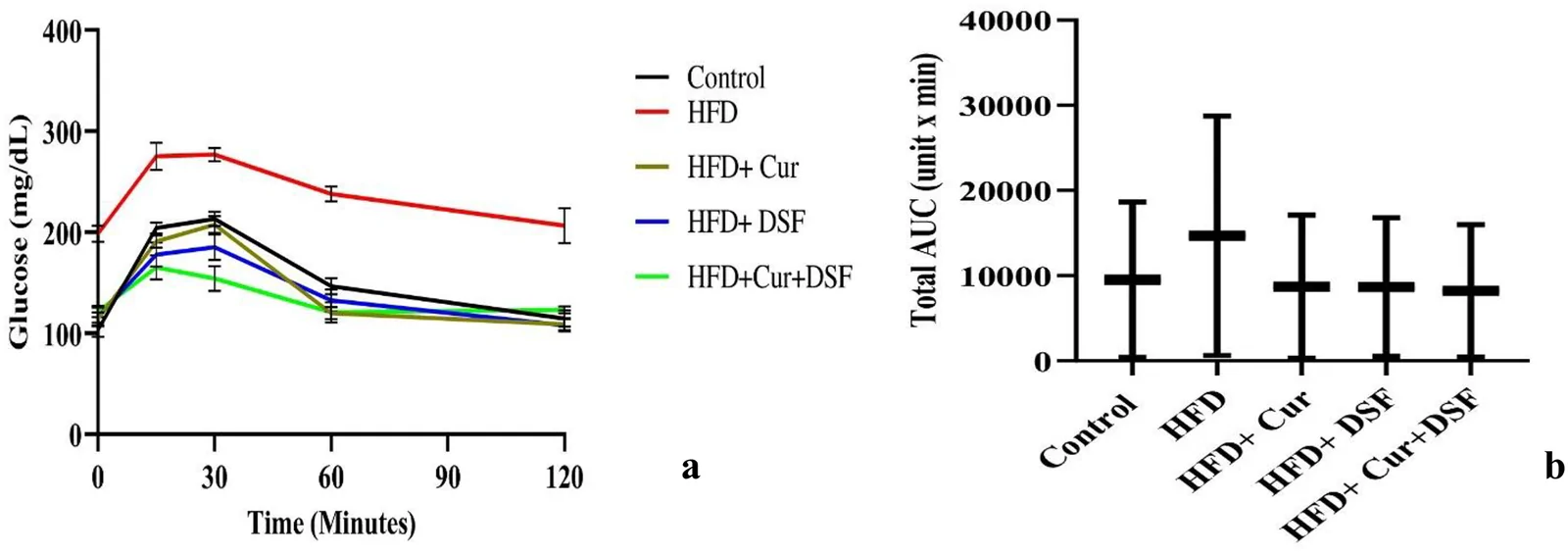
**(a)** Illustrates the oral glucose tolerance test (OGTT) in all the groups at the end of 12^th^ week, and **(b)** demonstrates the area under curve (AUC) for the same.

### Curcumin and disulfiram treatment mitigates HOMA-IR index, insulin and leptin resistance in HFD-induced obese mice

In the HFD group, the serum insulin level (**Figure 5a**) was 2.72 ± 0.42 ng/mL (p = 0.001), with a HOMA-IR value of 1.44 ± 0.03(p = 0.001), representing a nearly five-fold increased insulin level (0.73 ± 0.08 ng/mL), and with six-fold increase in HOMA-IR (0.23 ± 0.03) compared to the Control group. The serum leptin level was also significantly elevated in the HFD group (13.28 ± 0.71 ng/mL; p = 0.0010) (**Figure 5b**) relative to the Control group (4.27 ± 0.65 ng/mL), reflecting an expansion of adipocyte mass. Monotherapies with either Curcumin or Disulfiram significantly attenuated both insulin levels 1.09 ± 0.13 ng/mL, HOMA-IR 0.41 ± 0.04, and 1.36 ± 0.53 ng/mL, HOMA-IR 0.44 ± 0.02, respectively) and leptin levels (7.84 ± 0.82 ng/mL, p = 0.0010 and 7.38 ± 0.60 ng/mL, p = 0.0013, respectively). Notably, simultaneous treatment with both Curcumin and Disulfiram alongside HFD restored insulin levels almost entirely to the Control baseline (0.76 ± 0.13 ng/mL with HOMA-IR 0.24 ± 0.01) and markedly reduced leptin levels to 6.01 ± 0.45 ng/mL, demonstrating a superior therapeutic strategy to mitigate homeostatic hormonal resistance during diet-induced obesity.

**Figure 5:**
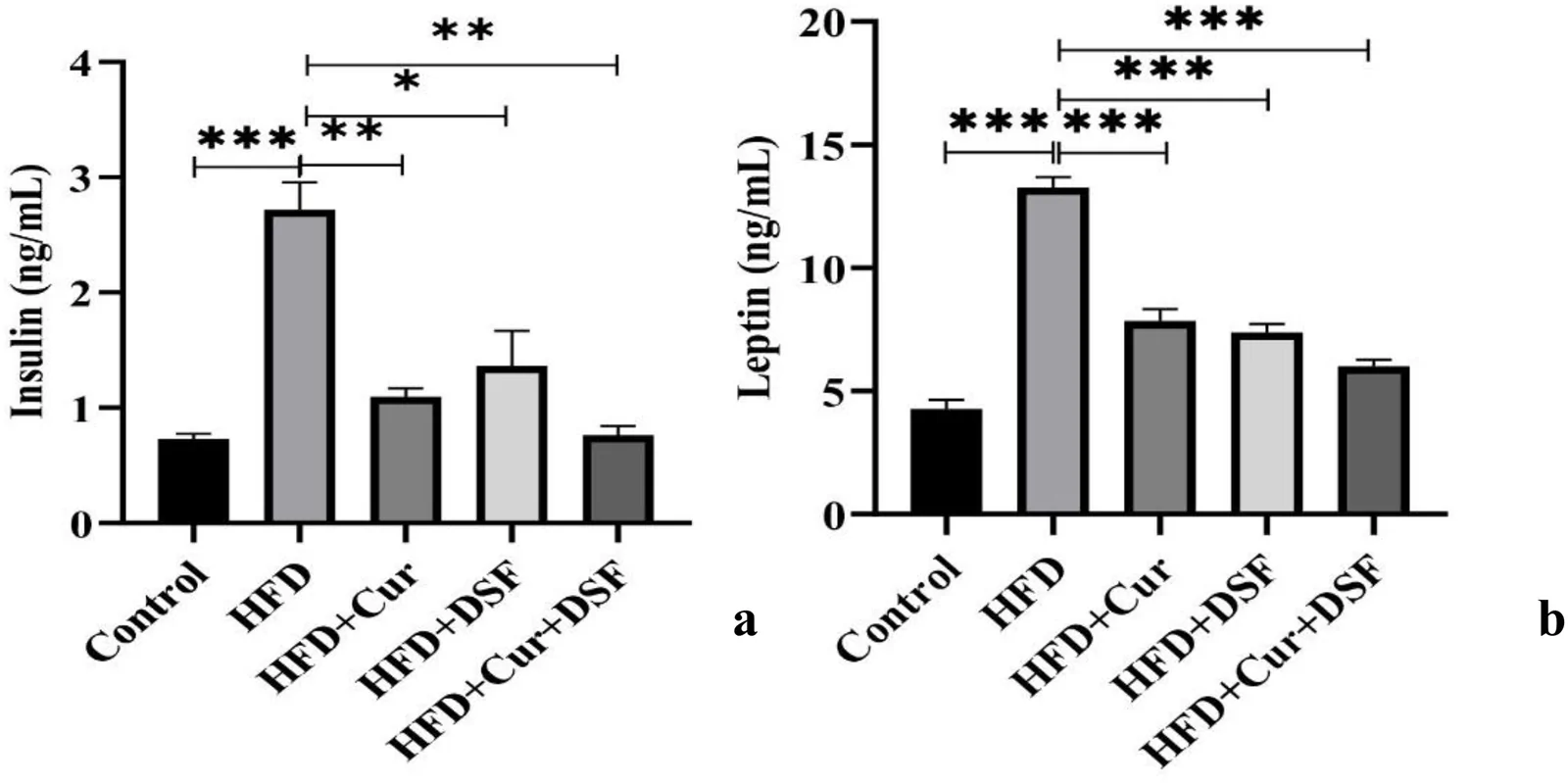
**(a)** Shows the Insulin level, and **(b)** shows the leptin level in various treated groups after the 12^th^ week of treatment. ***=p < 0.0001, ** =p < 0.01 and * =p < 0.05.

### Curcumin and disulfiram treatment improves serum lipid homeostasis in HFD-induced obese mice

The Triglycerides (120.54 ± 9.59 mg/dL) (**Figure 6a**), Cholesterol (208.23 ± 9.39 mg/dL) **Figure 6b**), LDL (68.53 ± 5.64 mg/dL) (**Figure 7a**), and HDL (141.87 ± 11.22 mg/dL) (**Figure 7b**), suggest that the HFD causes severely damaged lipid profiles, which was significantly low compared to the control Triglycerides, Cholesterol, LDL and HDL levels (96.20 ± 12.35 mg/dL, 92.20 ± 4.84 mg/dL; p = 0.0004, 23.93 ± 1.79 mg/dL; p = 0.0017 and 67.50 ± 3.42 mg/dL; p = 0.0032) respectively. Single treatment with Curcumin and Disulfiram with HFD groups shows significantly lower levels compared to the HFD groups. The simultaneous treatments with Curcumin and Disulfiram along with HFD, bring the levels equivalent to the control and show no significant difference between the Control and HFD + Cur +DSF group. This suggests that the combination treatment is more effective in preventing liver injury due to obesity compared to the single treatment.

**Figure 6:**
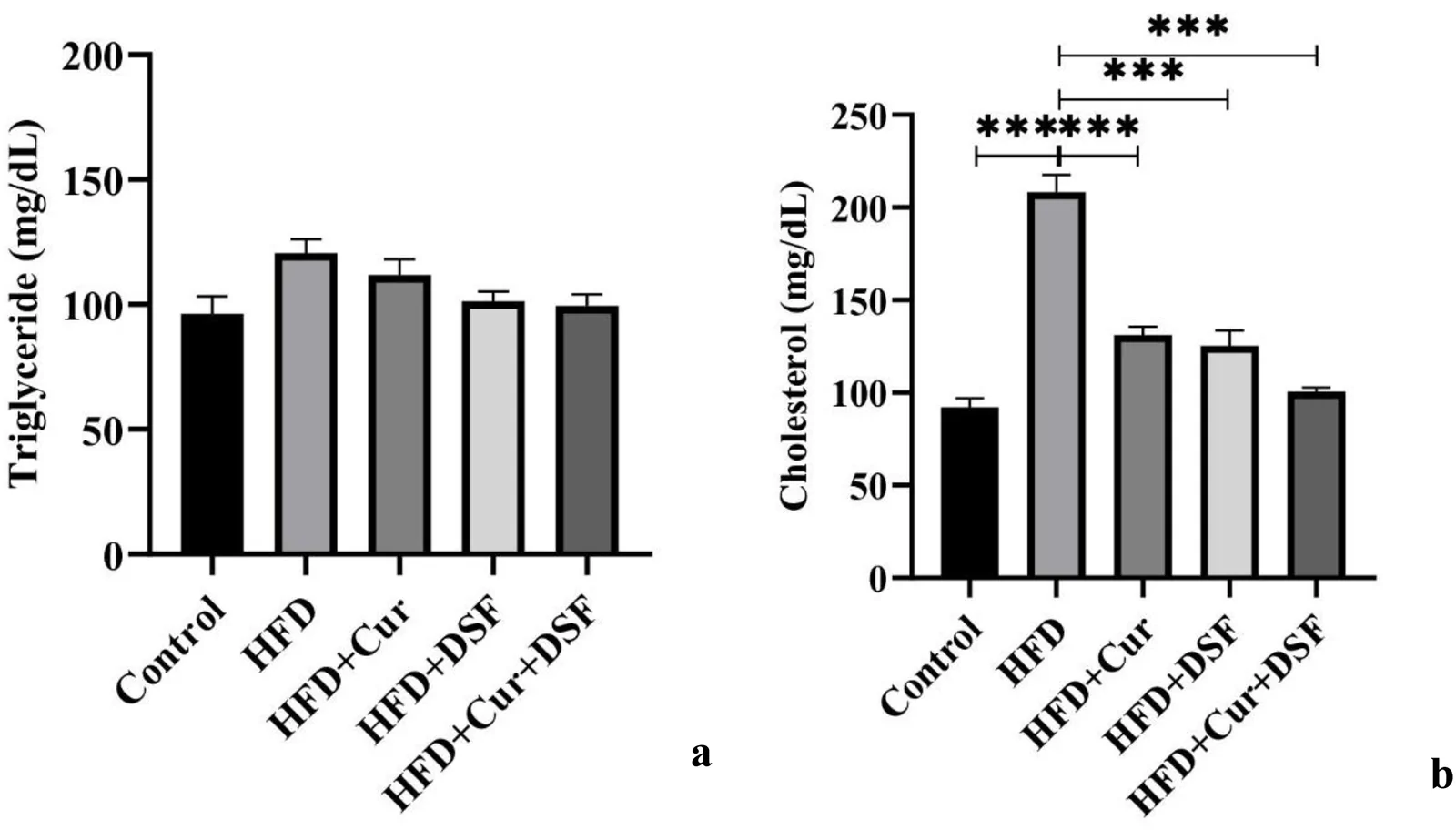
**(a)** Shows the Triglyceride level, and **(b)** shows the Cholesterol level in various treated groups after the 12^th^ week of treatment. ***=p < 0.0001. already provided in the data analysis section.

**Figure 7:**
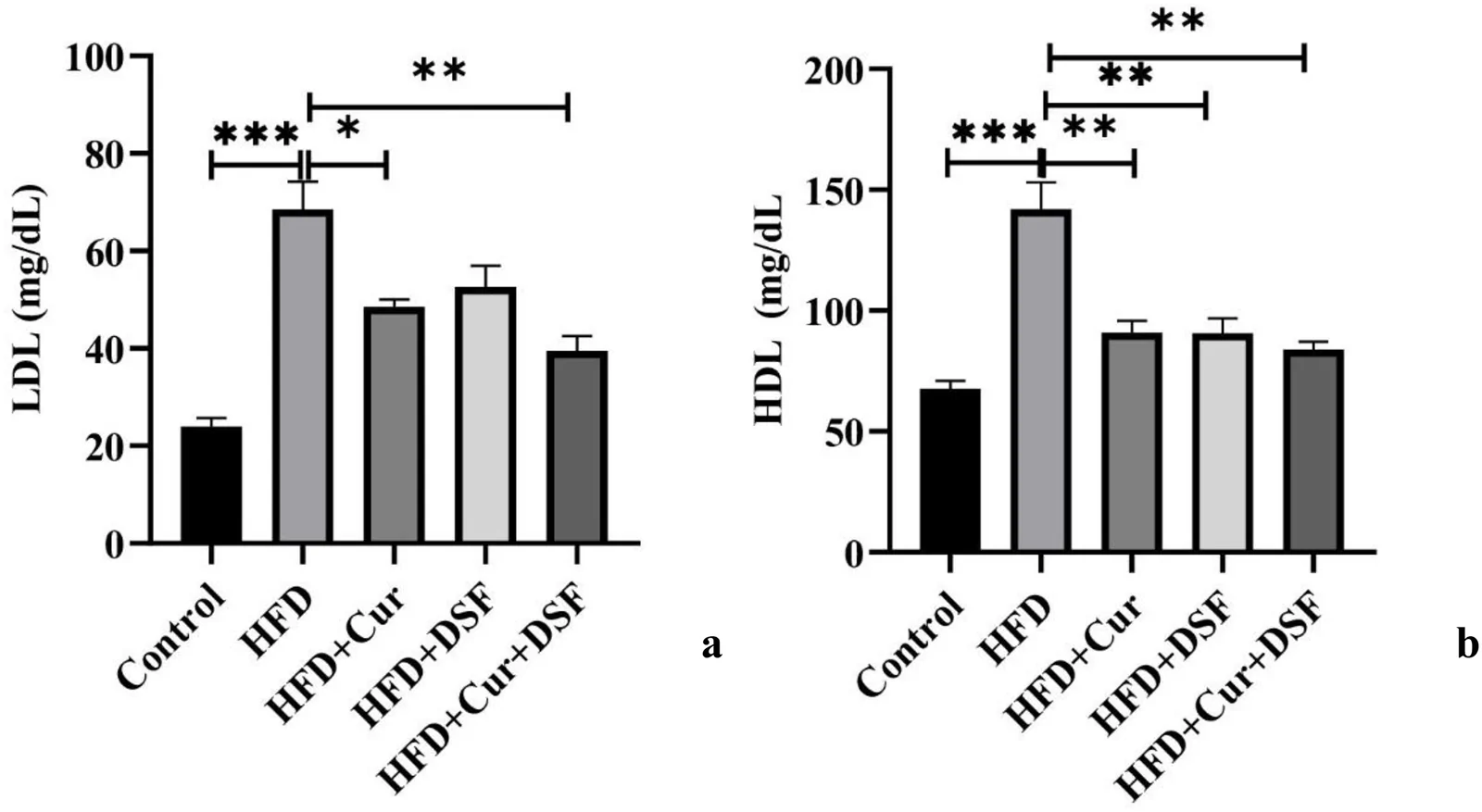
**(a)** Shows the low-density lipoprotein (LDL) level, and **(b)** shows the high-density lipoprotein **(HDL)** level in different treated groups after the 12^th^ week of treatment. ***=p < 0.0001, ** =p < 0.01 and * =p < 0.05

### Attenuation of hepatic injury biomarkers by curcumin and disulfiram treatment

The high-fat diet-fed group exhibited marked hepatocellular injury, characterized by a statistically significant elevation in serum liver enzymes. Serum Glutamic Pyruvic Transaminase (SGPT) levels (**Figure 8a**) rose from 42.96 ± 4.93 U/L in the control group to 87.28 ± 5.21 U/L in the HFD group (p = 0.0004). Similarly, Serum Glutamic Oxaloacetic Transaminase (SGOT) levels (**Figure 8b**) increased from 70.18 ± 6.17 U/L in the control group to 152.40 ± 6.71 U/L in the HFD group (p = 0.0010). Monotherapies with Curcumin (57.95 ± 2.30 U/L; p = 0.0082) or Disulfiram (59.19 ± 6.44 U/L; p = 0.0109) offered significant protection against this hepatic enzyme leakage. The combination treatment with Curcumin and Disulfiram (43.99 ± 3.06 U/L; p = 0.0005) successfully brought SGPT levels closest to the control baseline. For SGOT, the individual Curcumin and Disulfiram groups showed levels of 117.00 ± 5.30 U/L (p = 0.0030) and 117.68 ± 4.97 U/L (p = 0.0036), respectively, which were significantly lower than those of the HFD group. The combination treatment (105.93 ± 3.97 U/L; p = 0.0010) provided enhanced protection compared to either monotherapy.

**Figure 8:**
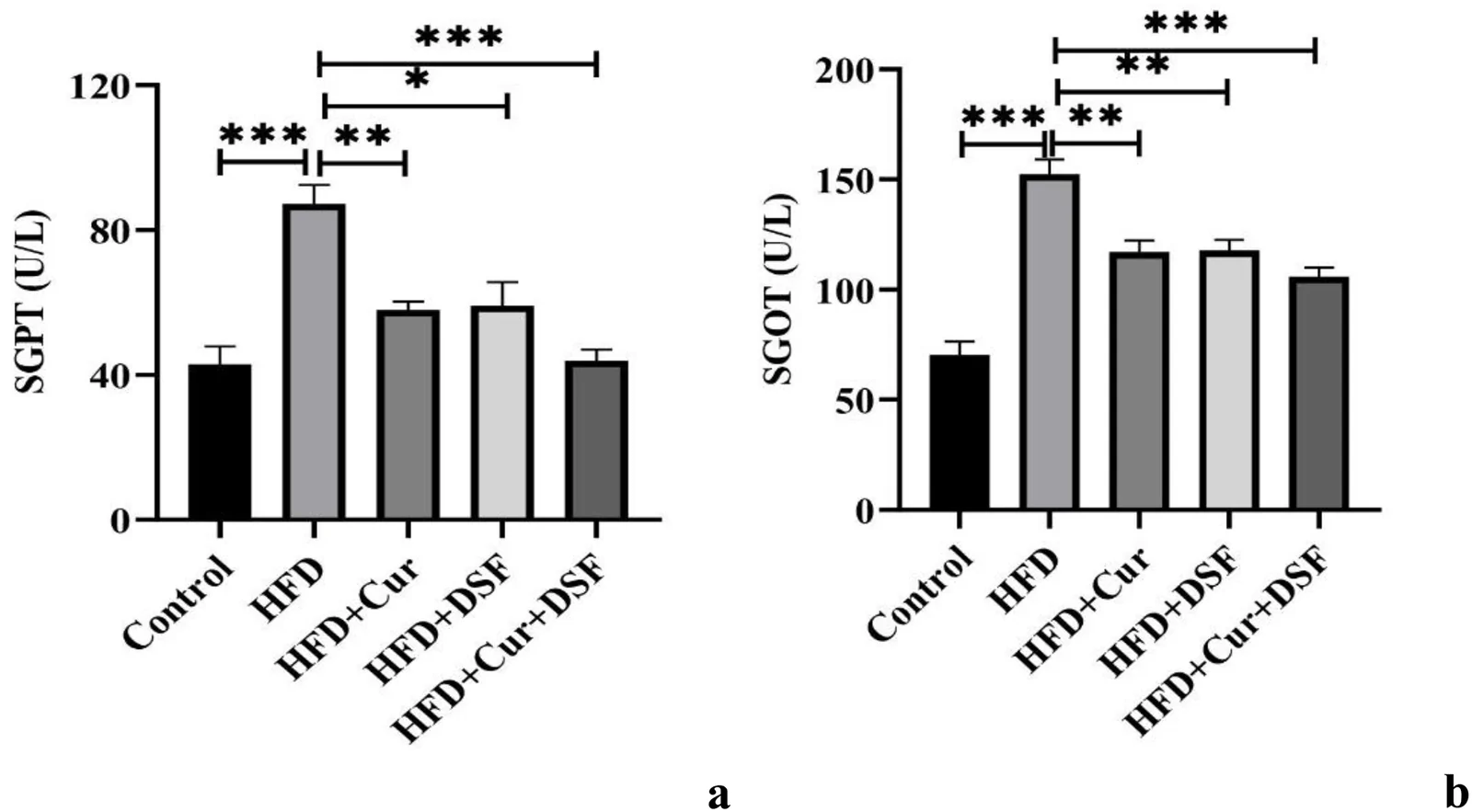
**(a)** shows the SGPT level, and **(b)** shows the SGOT level in different treated groups after the 12^th^ week of treatment. ***=p < 0.0001, ** =p < 0.01 and * =p < 0.05

Furthermore, liver histology at 200× magnification (**Figure 9**) revealed prominent infiltration of inflammatory cells (IfCs), ballooning of hepatocytes (H), and extensive accumulation of intracellular lipid droplets in the HFD group, confirming severe hepatocellular abnormalities. Individual treatments with Curcumin or Disulfiram reduced the number of lipid droplets and cleared inflammatory cell infiltration, confirming their anti-obesogenic properties. The combination treatment offered superior hepatoprotective effects compared to the monotherapies, restoring the hepatic architecture to a state highly similar to the control group. Analysis at 400× magnification (**Figure 10**) confirmed a distinct amelioration of hepatic steatosis-like pathology in the treated groups compared to the HFD group. However, mild hepatic sinusoidal (HS) dilations persisted, resulting from the vascular shifting initially caused by macrovesicular lipid droplets.

**Figure 9:**
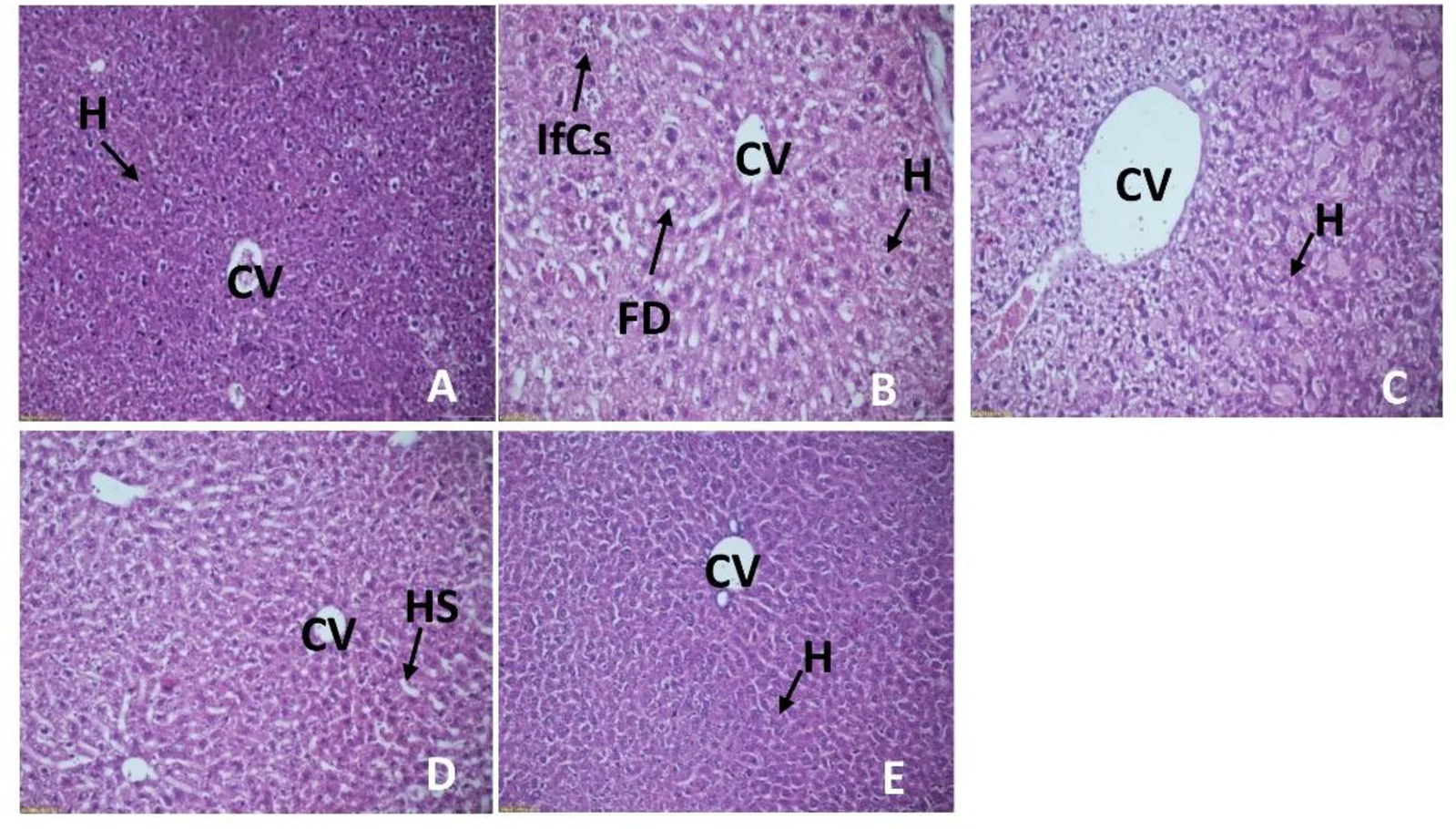
Shows the liver histology of different treated groups after the 12^th^ week of treatment at 200X magnification. **(A)** Control has normal hepatocytes, **(B)** HFD-fed shows infiltration of Inflammatory cells and fat droplets, **(C)** HFD-fed treated with Curcumin, **(D)** HFD-fed treated with Disulfiram and **(E)** HFD-fed treated with both Curcumin and Disulfiram.

**Figure 10:**
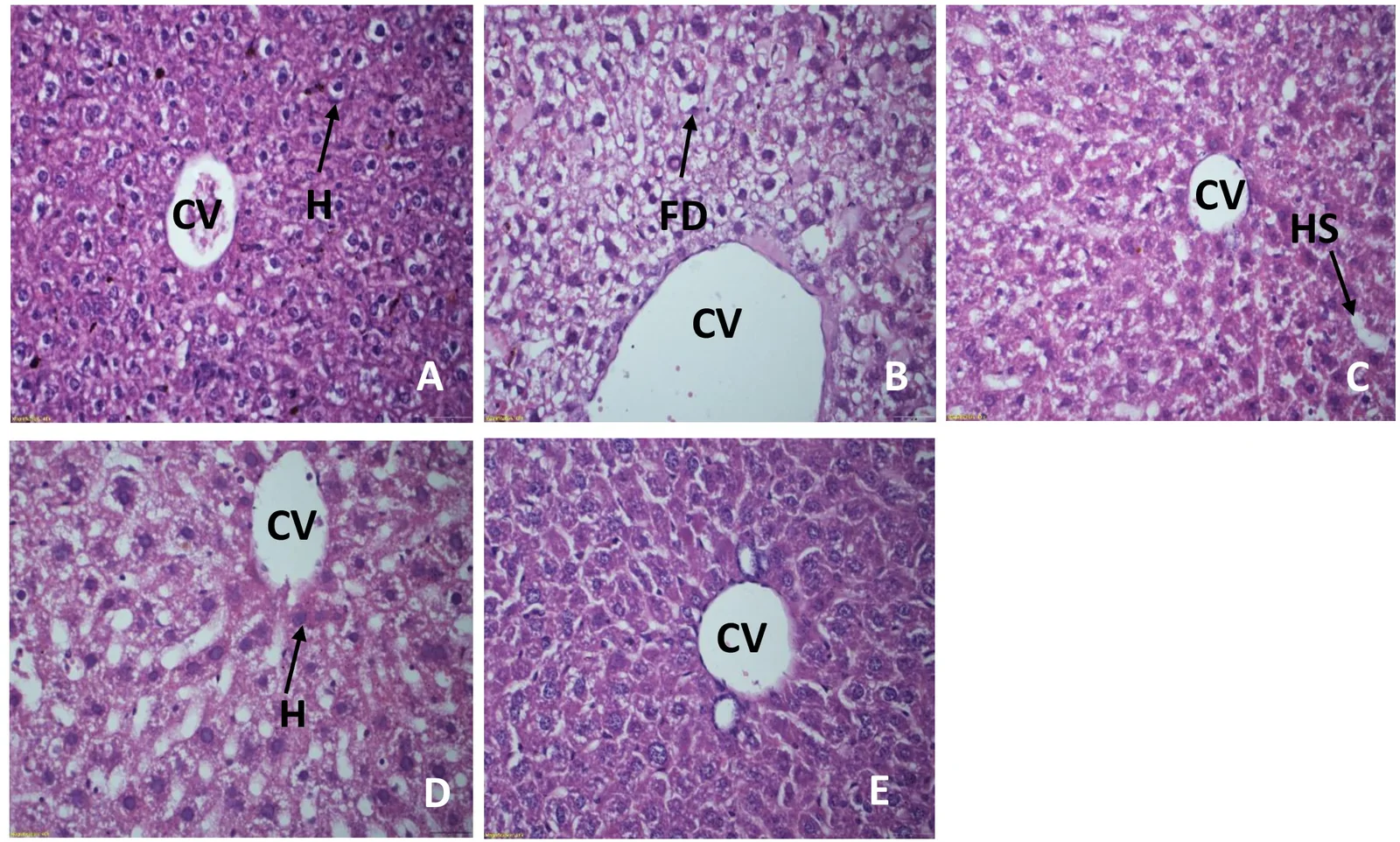
Shows the liver histology of different treated groups after the 12^th^ week of treatment at 400X magnification. **(A)** control has normal hepatocytes, **(B)** HFD-fed shows macrovesicular fat droplets, **(C)** HFD-fed treated with Curcumin, **(D)** HFD-fed treated with Disulfiram and **(E)** HFD-fed treated with both Curcumin and Disulfiram.

## Discussion

The present study demonstrates that while the combination of Curcumin and Disulfiram yields a substantial reduction in weight gain, the Curcumin monotherapy group achieves the most pronounced numerical weight reduction. Both the combination and Curcumin-treated groups exhibited suppressed feed consumption compared to the control and HFD-only groups, with Curcumin-treated mice showing an intake pattern highly similar to the combination cohort. These findings align with previous literature demonstrating that Disulfiram effectively prevents weight gain and reverses established obesity in rodents. Prior dose-dependent studies in rats comparing 100 mg/kg and 200 mg/kg body weight indicated that the higher dose accelerates weight reduction (13, 14). Consequently, the 200 mg/kg dose was selected for this study and successfully replicated significant weight loss. However, the toxicological aspect of the dose of disulfiram raised a question, as the FDA-approved dosing range for disulfiram in humans is 125–500 mg/day. Based on the commonly used body surface area conversion method for estimating human-equivalent doses, this dose corresponds to approximately 1,000 mg/day, which is much higher than the maximum allowed dose (500 mg). An adverse events study associated with disulfiram shows neurological and psychological issues (23). In parallel, the therapeutic efficacy of Curcumin observed here is supported by evidence that it reduces total body weight in HFD-induced obese mice by suppressing white adipose tissue inflammation (24). Furthermore, the post-treatment reduction of the Lee obesity index below 310 across all intervention groups validates the successful reversal of obesity (24). Regarding glycaemic control, the oral glucose tolerance test (OGTT) indicated that while both monotherapies improve glucose tolerance, the combination treatment clears the glucose load more rapidly and maintains the most stable homeostatic profile. In the untreated HFD group, the simultaneous elevation of serum insulin level and HOMA-IR value, an index of insulin resistance (25), indicates compensatory insulin hypersecretion and the development of insulin resistance due to HFD-induced obesity. Leptin resistance is associated with compromised leptin-mediated satiety signaling. Consistent with reports that Curcumin ameliorates insulin resistance in type 2 diabetic models (26), both single and combined treatments successfully downregulated insulin and leptin levels. Crucially, simultaneous administration revealed a distinct synergistic effect, bringing these metabolic hormones closest to baseline.

This synergistic protection extended to the lipid profile, where simultaneous Curcumin and Disulfiram administration reverted triglycerides, cholesterol, HDL, and LDL levels back to control baselines. At the tissue level, HFD-fed mice displayed severe histopathological alterations, including inflammatory cell infiltration, macrovesicular fat droplet accumulation, and hepatic sinusoidal distortion. These observations mirror established models of HFD-induced hepatic steatosis (12) and inflammatory infiltration (27), pathologies that directly cause the elevation of serum SGPT and SGOT. While Disulfiram has been shown to independently address hepatic steatosis and Curcumin is recognized for its hepatoprotective properties (26), the present study indicates that Curcumin monotherapy exerts a superior hepatoprotective effect compared to Disulfiram alone. Ultimately, the combination treatment provided the most comprehensive therapeutic benefit, driving a marked reduction in SGPT and SGOT levels and restoring the structural integrity of the liver closest to the control group.

## Conclusion

Simultaneous administration of Curcumin and Disulfiram alongside a high-fat diet significantly prevents diet-induced obesity, maintaining mice at a body weight comparable to those on a normal diet. This combination provides a distinct synergistic benefit by preventing HFD-induced glucose intolerance and markedly reducing the total glycaemic burden, as reflected by the decreased Area Under the Curve (AUC). In contrast, the untreated HFD group develops a classic metabolic paradox: simultaneous elevations in glucose, insulin, HOMA-IR index and leptin levels confirm the onset of severe systemic insulin and leptin resistance. The combination treatment successfully reverses this pathology, returning these hormonal and glycaemic parameters to control baselines. Furthermore, the combination synergistically downregulates abnormal lipid profiles and elevated liver enzyme levels, mitigating hepatic stress. Ultimately, the co-administration of Disulfiram and Curcumin exerts an enhanced anti-obesogenic effect by reducing food intake, improving glucose metabolism via the reduction of adipocyte mass, and downregulating metabolic hormones. This dual approach demonstrates a robust preventive profile against diet-induced obesity and type 2 diabetes.

### Current limitations and future perspective

While research highlights the therapeutic potential of Disulfiram and Curcumin, several limitations remain for future study. The pharmacological profile of Disulfiram requires comprehensive toxicological assessment to fully understand its safety profile in various models. Additionally, the clinical utility of Curcumin is often limited by its metabolic properties, which affect how much of the compound remains active in the body. Future investigations should focus on exploring advanced delivery methods to improve bioavailability.

## Data Availability

The original contributions presented in the study are included in the article/supplementary material. Further inquiries can be directed to the corresponding author.
